# Supplementary material for: Structural disconnectome mapping of cognitive function in poststroke patients
Source: Brain Behav. 2022 Jul 21;12(8):e2707. doi: 10.1002/brb3.2707 (PMC9392540; doi:10.1002/brb3.2707)
Supplement: Supplementary file 1 — TABLE S1 Summary of association between MoCA performance, and lesion size, both with and without 1 outlier FIGURE S1 Association between lesion size and MoCA performance, after removal of one outlier FIGURE S2 Association between lesion size and MoCA performance, for the full sample [file BRB3-12-e2707-s001.docx]

**Supplementary Material**

Supplementary Table 1

|  | Beta estimate | t-value | p-value |
| --- | --- | --- | --- |
| Without outlier | | | |
| Lesion Size | -5.630e-05 | -1.710 | 0.0904 |
| Lesion Count | -1.851e-01 | -1.254 | 0.2130 |
| Outlier included | | | |
| Lesion Size | -3.464e-05 | -2,96 | **0.00385** |
| Lesion Count | -2.131e-01 | -1,5 | 0,1364 |

*Supplementary table 1: Summary of association between MoCA performance, and lesion size, both with and without 1 outlier.*

Supplementary Figure 1


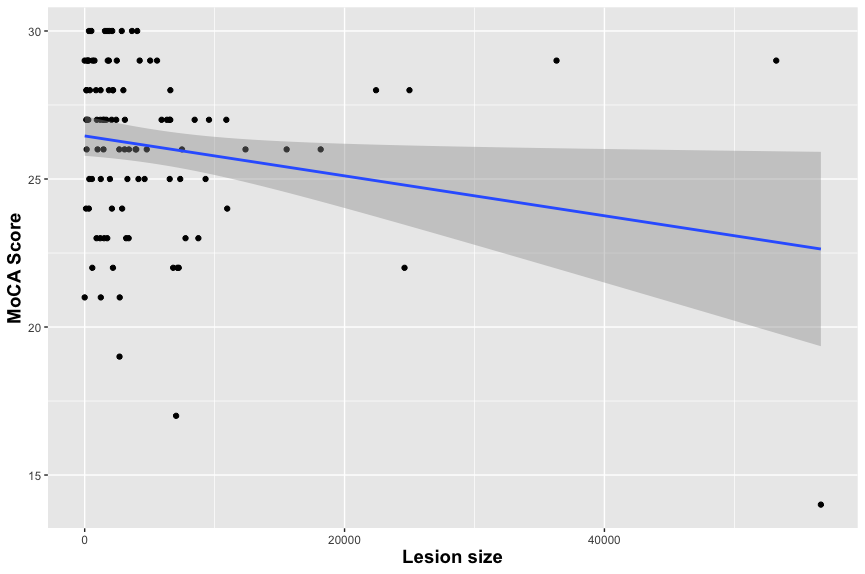


*Supplementary figure 1: association between lesion size and MoCA performance, after removal of one outlier.*

Supplementary Figure 2


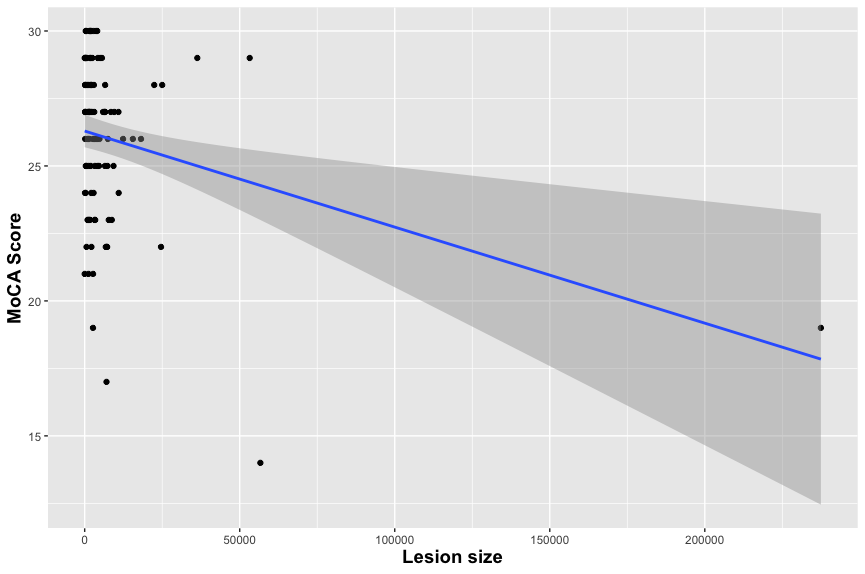


*Supplementary figure 2: Association between lesion size and MoCA performance, for the full sample.*
